# Statistical Optimization and *In Vitro* Evaluation of Metformin Hydrochloride Asymmetric Membrane Capsules Prepared by a Novel Semiautomatic Manufacturing Approach

**DOI:** 10.1155/2013/719196

**Published:** 2013-12-08

**Authors:** Venkatesh Teja Banala, Bharath Srinivasan, Deveswaran Rajamanickam, Basavaraj Basappa Veerbadraiah, Madhavan Varadarajan

**Affiliations:** ^1^Department of Pharmaceutics, M. S. Ramaiah College of Pharmacy, M. S. R. Nagar, MSRIT Post, Bangalore 560 054, India; ^2^Department of Pharmacognosy, M. S. Ramaiah College of Pharmacy, Bangalore 560 054, India

## Abstract

Asymmetric membrane capsules (AMCs) are one of the novel osmotic delivery devices which deliver a wide range of drugs in controlled manner. In the present work, we developed and validated a semiautomatic process by fabricating a hydraulic assisted bench top model for manufacturing AMCs. The capsule walls of AMCs were prepared by dip coating phase inversion process using cellulose acetate butyrate (CAB) as coating polymer and propylene glycol (PG) as plasticizer and pore former. The comparative examination of physical parameters confirmed the consistency, efficiency, and reproducibility of the semiautomatic process over the manual procedure. The SEM studies revealed a thin dense region supported on a thicker porous membrane of the capsule shells. Formulations of AMCs were prepared based on a 2^3^ full factorial design using metformin hydrochloride as the model drug. The effect of formulation variables such as concentration of PG and levels of fructose and potassium chloride were studied on the *in vitro* drug release using Design-Expert 8.0.2 (USA) software. From the *in vitro* release studies, it was observed that the concentration of pore former and level of osmogents had a direct effect on the drug release. From the validation studies of the optimized formulation (OPT) with the predicted response, it was observed that the drug release was independent of pH and agitation intensity but dependent on osmotic pressure of the dissolution medium. The OPT followed controlled zero-order release kinetics over a period of 13 h.

## 1. Introduction

Controlled release drug delivery systems have been the research hot spot for the formulation scientists from the last few decades. These delivery systems became popular due to their sustained release and reduction in dosage frequency which leads to the patient compliance. A number of design approaches were available to control or modulate the drug release from a dosage form. The majority of sustained release dosage forms come under the category of matrix, reservoir, or osmotic systems. The application of osmotic pressure for drug delivery was extensively studied and explained by Santus and Baker [1] as the most acceptable approach to achieve the zero-order kinetics.

Asymmetric membrane capsules (AMCs) are one of the single core nondisintegrating osmotic controlled systems consisting of drug filled in water insoluble polymer shells [2]. Since the capsule is made of water insoluble semipermeable polymer, the drug release is controlled by osmotic pressure as a major contribution. The *in vitro* release rate of a drug from an AMC depends on the capsule shell composition as well as the fill (core) formulation. For a given shell composition, the release depends on osmotic pressure (solubility) of the core ingredients and, for a given core composition, the release is dependent on the capsule shell permeability [3].

The development of AMCs involves several interrelated process parameters which makes it a complex process. In 1999, Thombre et al. proposed a semiautomatic pilot scale manufacturing setup for the development of AMCs [4]. But due to its high cost and maintenance of the setup, it was not suitable for initial stages of the formulation development. Till date, no reports were mentioned in the literature, for the development of AMCs by lab scale mechanical manufacturing process. To achieve this, in the present work we demonstrate the fabrication of a semiautomated bench top model for the development of AMCs with consistent quality, for the full scale formulation development. The fabricated instrument has been validated with cellulose acetate butyrate (CAB) and metformin hydrochloride as a model drug.

Metformin hydrochloride is a highly water soluble antidiabetic drug from the biguanide class. It has been reported that the absolute bioavailability of metformin when given orally is 50–60% with biological half-life of 4.5–6 h. Being an ideal drug candidate for controlled release, in the present study an osmotic controlled delivery system using AMCs was planned to deliver metformin hydrochloride for a prolonged period of time [5, 6]. *In vitro* release of metformin hydrochloride was optimized by 2^3^ full factorial design using Design-Expert 8.0.2 software.

## 2. Materials and Methods

### 2.1. Materials

Metformin hydrochloride was procured from Micro Labs (Bangalore, India). Cellulose acetate butyrate (CAB) was purchased from Hi-Media Laboratories Pvt. Ltd. (Mumbai, India). Fructose was obtained from Merck Specialities Pvt. Ltd. (Mumbai, India). Potassium chloride was procured from Qualigens Fine Chemicals (Mumbai, India). All other chemicals and reagents were of analytical grade. Solvents of reagent grade and double distilled water were used in all experiments.

### 2.2. Design Description of the Fabricated Lab Equipment for the Manufacture of Asymmetric Membrane Capsule Shells

**Figure 1:**
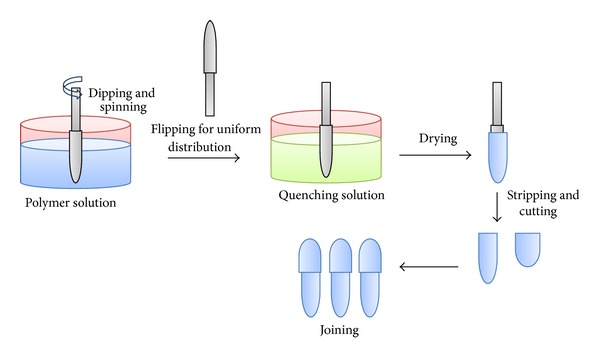
General scheme showing the manufacturing of AMCs.

Semiautomatic hydraulic assisted bench top equipment was designed and fabricated for the manufacturing of AMCs. The processing steps simulate simple hard gelatin capsule shell preparation like dipping, spinning, flipping, drying, and so forth, as shown in Figure 1.

**Figure 2:**
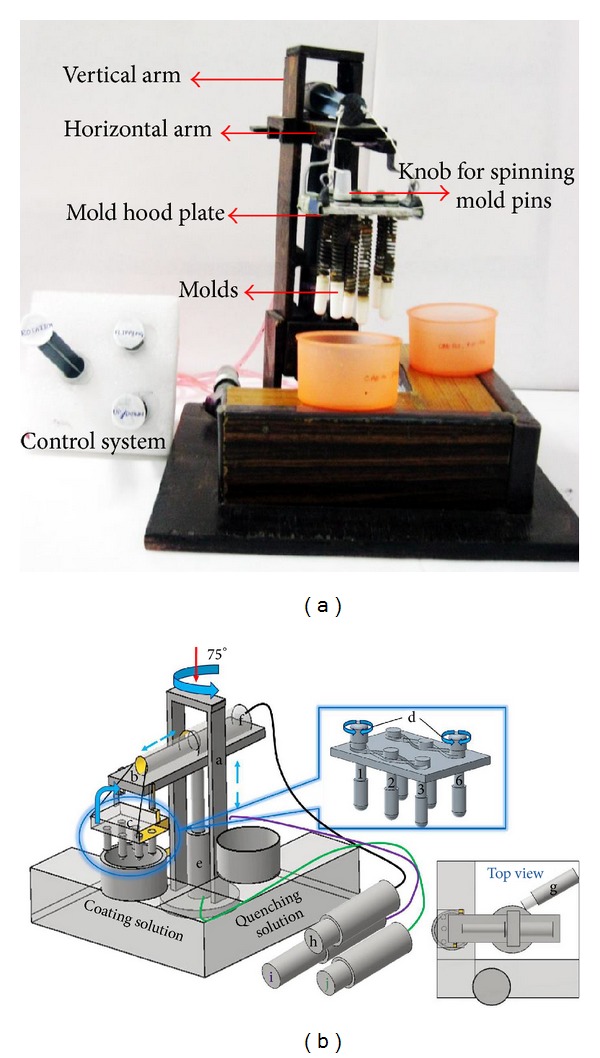
(a) Original image (left) of the fabricated equipment with labeled parts; (b) 3D sketch of the fabricated instrument (right) with top view showing the alignment of plunger connected for angular rotation; parts: (a) vertical arm, (b) horizontal arm, (c) mold hood plate with mold pins, (d) two knobs on mold plate for spinning, (e) syringe plunger-1 connected to horizontal arm for up/down movement, (f) syringe plunger-2 connected to mold plate for flipping movement, (g) syringe plunger-3 connected to disc for angular rotation and controls (h, I, and j) connected to mold hood and vertical and horizontal arm.

The skeleton of the equipment was designed by modifying the mechanical robotic arm, which works on the principle of hydraulic pressure. The required hydraulic pressure was obtained by using 20 mL and 15 mL water filled polypropylene syringes connected with pressure resistant rubber tubing's. The forward movement of one plunger results in the backward movement of the corresponding plunger due to induced pressure flow of water. This mechanism helps in the control of arm in two-dimensional movement. The original fabricated instrument was shown in Figure 2(a). A wooden arm was made in such a way to move in two-dimensional axes which facilitates the movements like up/down and angular rotary motion.

The 3D sketch has been designed using Autocad inventor fusion 2013 software for clear and easy understanding of the instrument (Figure 2(b)). The equipment consists of two arms, vertical arm, and horizontal arm. The horizontal arm was connected to the vertical arm with the help of a plunger of the syringe which facilitates the up and down movements; the vertical arm can be rotated in certain angle with the help of disc connected to one more syringe.

A removable mold setup was connected to the horizontal arm which holds the plate containing mold pins. The entire mold setup was connected to one more syringe on the top of horizontal arm to facilitate inversion of mold plate.

The mold plate was designed in such a way to remove and reinsert a new plate for every fresh batch. For this equipment, a mold plate was used which can accommodate three pairs of mold pins at a time which can be removed and replaced with a new set of mold pins each time.

#### 2.2.1. Design Specifications of the Molds and Mold Plate

**Figure 3:**
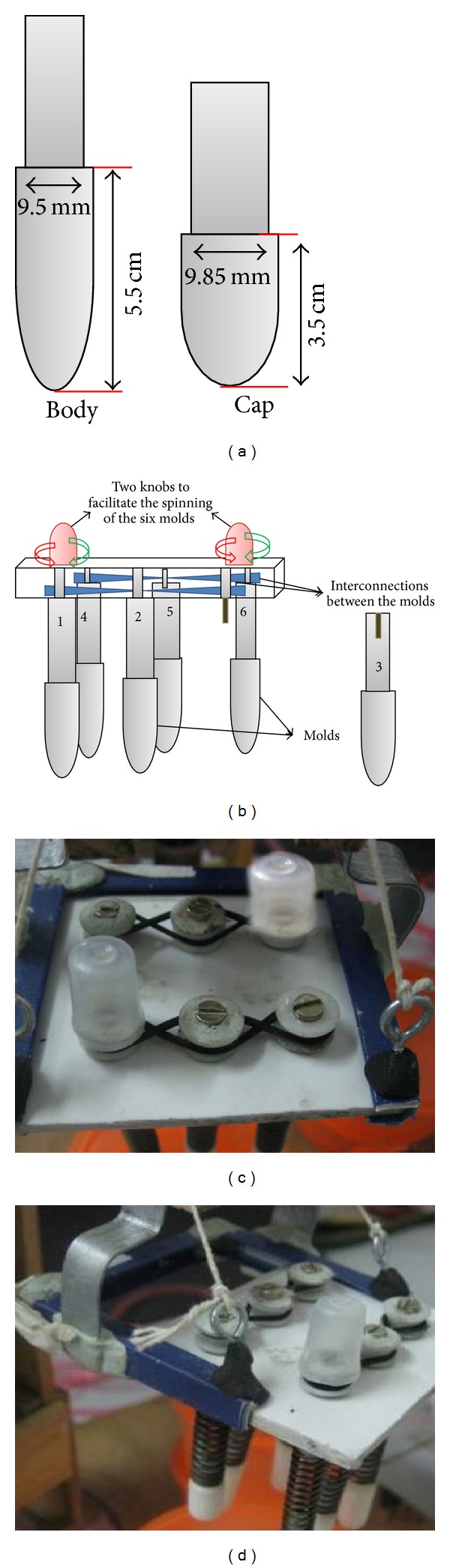
(a) Dimensions of the cap and body. (b) 2D sketch showing the alignment of the mold pins, (c) original image of the mold plate and (d) Rack provided to withdraw mold plate.

The capsule molds were fabricated, separately for the cap and body using Teflon to facilitate smooth and easy removal of the dried capsule shells without any prior lubrication. As the drying conditions required are below 50°C, Teflon molds were found to be most suitable and convenient for the manufacturing process. The molds were fabricated in such a way to snugly fit into cap and body. The dimensions of the cap and body in length : diameter ratio are as follows 35 : 9.85 and 55 : 9.5 mm, respectively, as shown in Figure 3(a).

The spinning of the mold pins can be facilitated by the two knobs which are arranged diagonally on the mold plate (5 × 7.5 cm). Each knob was connected and interlinked with other two disc plate pins by which rotating one knob will facilitate the spinning of all three mold pins in the same row as shown in Figures 3(b), 3(c), and 3(d). So, the two knobs facilitate the spinning of six mold pins in either clockwise or anticlockwise direction according to the requirement.

#### 2.2.2. Working Process of the Fabricated Equipment

**Figure 4:**
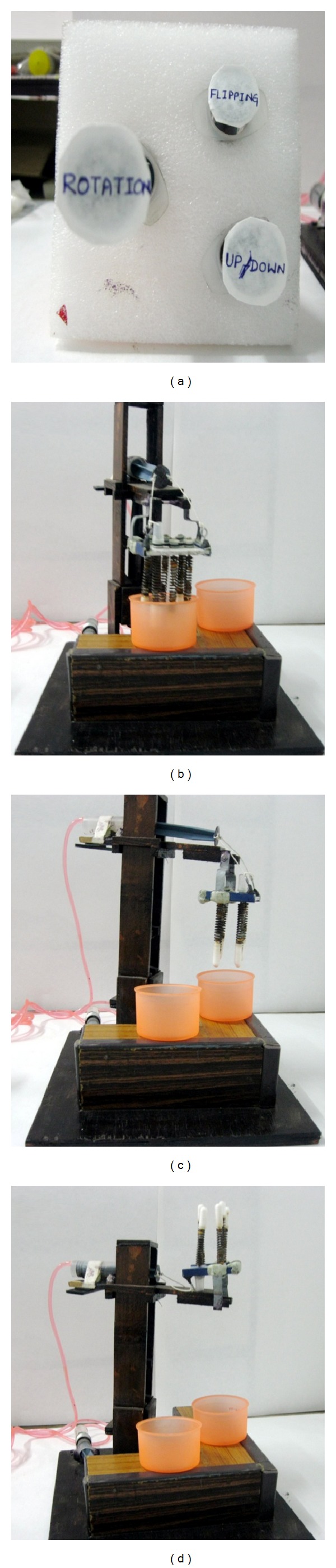
Original images showing the (a) control system, (b) up/down movement, (c) angular rotation, and (d) flipping of the mold hood.

The designed equipment assists two dimensional motion, that is, up/down and angular rotation, by three separate controls (up/down, rotation, and flipping) as shown in Figure 4(a). These three controls respectively operate the movements of horizontal arm, vertical arm, and mold plate as shown in Figure 4(b), 4(c), and 4(d). The vertical arm can move at an angle of maximum 75° in anticlockwise direction.

#### 2.2.3. Development of Manual and Semiautomatic Method for Manufacture of AMCs

AMCs were prepared by the wet phase inversion process, in which the polymeric membrane was precipitated on Teflon mold pins by dipping in coating solution of CAB dissolved in acetone: ethanol mixture (3 : 7 *v/v*). After withdrawing from the coating solution, the mold pins were allowed to air-dry for 30 s followed by quenching in 5% *v/v* of aqueous propylene glycol for 3 min to get asymmetric shell. After removing from quench bath, the body and cap from the molds were stripped according to the length of conventional hard gelatin capsules and dried at ambient temperatures (30–35°C) for 6 h. Different concentrations of CAB (10%, 12%, 14%, and 16% *w/v*) and propylene glycol (10, 15, and 20% *w/v*) were prepared as per the Table 1. In the semiautomatic process same manufacturing procedure was followed using fabricated equipment. With an aim of developing AMCs of uniform thickness an optimized formulation CAB-12 was selected and validation of the instrument was performed to check the consistency and reproducibility of the capsule shells.

### 2.3. Characterization of AMCs

#### 2.3.1. Thickness and Weight Variation

The prepared asymmetric capsules of CAB at different concentrations were cut transversely as strips and the thickness was measured with the help of screw gauge and the average weight of 20 capsules of the individual batches was determined. The body and cap of the capsule shells were trimmed to appropriate size and snugly fixed each other before measurement.

#### 2.3.2. Diameter

The diameters of the cap and body of the capsule shells (*n* = 10) were determined individually for all the formulations of CAB using vernier calipers and the mean diameter was calculated.

#### 2.3.3. Osmotic Release Study

To confirm the osmotic release mechanism, the capsule shells of optimum concentration (CAB-12) were selected. The capsule shells were filled with water soluble dye erythrosine along with osmogent (potassium chloride and fructose), sealed with 12% *w/v* of CAB. Then the capsules were suspended separately in beakers containing 250 mL of water and sodium chloride solution (10% *w/v*). The capsules were observed visually for the release of colored dye [7, 8].

#### 2.3.4. Scanning Electron Microscopy

AMCs of CAB-12 with different concentrations of PG (10%, 15%, and 20%) were examined for their outer dense and inner porous morphology by scanning electron microscope (JEOL 840 A, Tokyo, Japan). Membranes were air-dried for 8 h and stored between sheets of wax paper in a desiccator before examination. The asymmetric membrane samples were sputter coated for 5–10 min with gold using the fine-coat ion sputter (DMX-220A, Beijing, China) at 50 mA and examined under SEM at suitable magnification.

#### 2.3.5. Validation of the Fabricated Equipment

Validation of the fabricated equipment was performed by comparative evaluation with the manual process in thickness and weight variation of individual molds.

### 2.4. Preparation and Characterization of Plain and Asymmetric Membranes

Fourier transform infrared spectroscopy (FTIR) and water vapor transmission studies were carried out to check the difference between plain and asymmetric Membranes (AMs). CAB-12 formulations of AMCs with different concentrations of PG were casted on glass petri plates by maintaining the same conditions used in the capsule manufacturing process except quenching step in the preparation of plain membranes.

#### 2.4.1. FTIR Spectral Studies

FTIR spectra of the plain and asymmetric films were recorded with Shimadzu 8400S, Japan. The spectra were collected as the average of 20 scans with a resolution of 4 cm^−1^, from 4000 to 400 cm^−1^ in transmission mode.

#### 2.4.2. Water Vapor Transmission Rate (WVTR)

The WVTR was measured according to ASTM E96-80, modified by McHugh and Krochta [9]. Membrane specimens (ø 15 mm) were placed to cover glass vials of identical dimensions containing saturated solution of calcium chloride. Then the vials were placed in an environmental chamber (Tempo Instruments, India) for 72 h at 30°C, where the relative humidity (RH) was maintained at 85% with the help of hygrometer. The weights of the glass vials were recorded at regular time intervals to calculate water vapor transmission rate and an average value was obtained.

The water vapor transmission rate (WVTR) was calculated according to

(1) $$\begin{array}{c} \text{WVTR}=\frac{w\times x}{t\times A}\;=\;\text{g}\cdot \text{mm}\cdot 24\;{\text{h}}^{-1}\cdot \text{c}{\text{m}}^{-2}, \end{array}$$

where *w* is the weight change in grams, *A* is the permeation area, and the term *x*/*t* was calculated by linear regression from the points of weight gain and time, during constant rate period.

### 2.5. Formulation of AMCs Containing Metformin Hydrochloride

#### 2.5.1. Experimental Design

2^3^ full factorial design was used as an experimental design to optimize and evaluate the AMCs filled with metformin hydrochloride. The concentration of propylene glycol (*A*) and amount of osmogents, potassium chloride (*B*), and fructose (*C*) were selected as independent variables. Each variable was set at high level and low level based on the results of preliminary experiments. Time taken for the 100% drug release (*t*_100%_) was taken as response parameter. The actual and coded values of different variables are given in Table 2 and the eight formulations were prepared according to the design as shown in Table 3.

#### 2.5.2. *In Vitro* Drug Release

*In vitro* drug release studies of metformin hydrochloride AMCs were performed using the USPXXIII Type-I basket type dissolution apparatus (Labindia DS8000, India) for 12 h using 900 mL of distilled water as dissolution medium with an agitation speed of 100 rpm at 37 ± 0.5°C. 5 mL of sample was withdrawn at periodic time intervals and the same volume of fresh media was replaced to maintain sink conditions. The collected samples were diluted appropriately by fresh media and analyzed UV spectrophotometrically at *λ*_max⁡_ = 233 nm. The cumulative amount of drug released at each time point was plotted against time.

#### 2.5.3. Kinetics of Drug Release

To describe the kinetics of drug release from drug delivery system, various mathematical models have been proposed, namely, zero-order, first-order, Higuchi model, [10] and Hixson-Crowell cube root law [11]. The best fit model was selected based on highest linearity of the data when incorporated in PCP Disso Software (PCP Disso Version 2.08 Software, Pune, India).

#### 2.5.4. Statistical Analysis

Design Expert 8.0.2 (Stat-Ease, Inc., USA) was used for the analysis of each variable effect on the designated response. Pareto charts were made for the analysis of each response coefficient contribution for its statistical significance. Quantitative and qualitative contribution of each variable on the response factor (*t*_100%_) was analyzed. The significant response polynomial equation generated by Design Expert was used to validate the statistical design. Possible interactions between *AB*, *BC*, and* CA* were studied and surface plots were generated to predict the simultaneous effect of each variable on the response factor.

### 2.6. Selection and Validation of Optimized Formulation

Optimized formulation (OPT) was selected based on 100% cumulative drug release at 12 h, with coefficient of determination (*r* ^2^) for zero-order release with a good desirability. The OPT was validated by comparing *in vitro* drug release with the predicted response, and the effect of environmental factors like pH, agitation intensity, and osmotic pressure on the drug release was determined.

### 2.7. Effect of pH and Agitation Intensity

The effects of media pH and agitation rate on the drug release were investigated for the OPT using different media (distilled water, 0.1 N HCl, phosphate buffer pH 6.8 and 7.4) at 100 rpm, as well as in varied agitation intensities (50, 100, and 150 rpm) by using distilled water as dissolution media, maintaining 900 mL as the volume at 37 ± 0.5°C.

### 2.8. Effect of Osmotic Pressure

In order to confirm the mechanism of drug release, release studies of the optimized formulation (OPT) were conducted in media of different osmotic pressure. Dissolution was carried out in 900 mL of distilled water (0 atm. osmotic pressure) and 2.4% *w/v* of magnesium sulphate (6 atm. osmotic pressure) in alternative 3 h at 100 rpm and the release profile was analyzed UV spectrophotometrically at *λ*_max⁡_ = 233 nm.

## 3. Results and Discussion

In the present study a semiautomatic lab model capsule shell manufacturing equipment was designed and fabricated to produce an output capacity of 80–100 units per day. CAB AMCs were prepared by phase inversion technique of dip coating process manually using polymer concentration between 10 and 16% *w/v* using propylene glycol (PG) of 10, 15, and 20% *v/v* as plasticizer and pore forming agent.

The physical characteristics of the capsules shells of different formulations were analyzed for reproducibility, uniformity, and intactness between body and cap. The AMCs of CAB-10 were found to be very thin and delicate with poor mechanical strength, due to lower concentration of polymer. Capsule shells of good mechanical strength were formed in higher concentrations (CAB-12, CAB-14, and CAB-16), but the rigid film with poor intactness of cap and body made CAB-14 and CAB-16 formulations not suitable for the capsule preparation. Thus, CAB-12 formulation with varied concentration of the plasticizer (PG) was selected for the formulation development.

### 3.1. Thickness and Weight Variation

**Figure 5:**
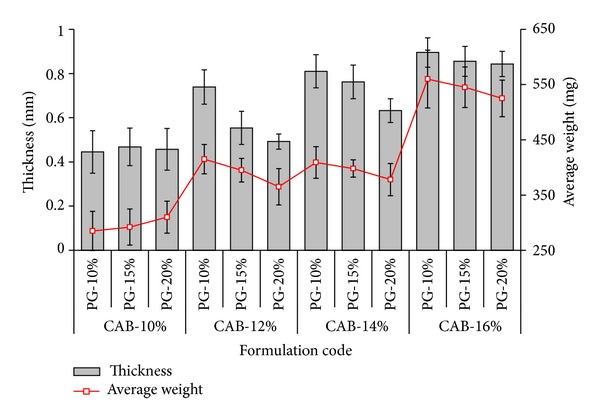
Thickness (*n* = 3) and average weight (*n* = 20) of different formulations CAB-AMCs.

The data of the thickness and weight variation clearly demonstrated the cumulative effect of concentration of the polymer and plasticizer (Figure 5). It was observed that polymer concentration had a positive effect whereas PG concentration had a negative impact on the thickness and average weight of the AMCs. The weight and thickness of the capsule shells were found to be decreased with the increase in plasticizer at an individual concentration of the polymer. This may be due to the decrease in thickness with the increase in spreading efficiency and plasticity of membrane [12].

### 3.2. Diameter

**Figure 6:**
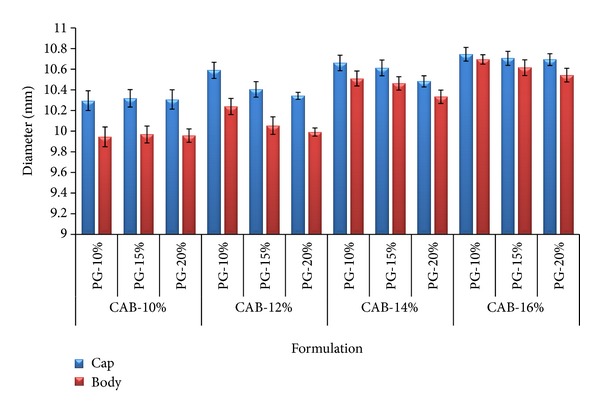
Comparative bar graph showing the outer diameter of the cap and body of the capsules (*n* = 10).

Increase in the diameter was observed as a proportional factor to the concentration of the polymer as shown in Figure 6. The formulation CAB-10 was found to be delicate and the formulations with higher polymer concentrations (CAB-14 and CAB-16) were found to be poor intact and flexible with rigid structure. Among all formulations, CAB-12 AMCs were found to be better reproducible with intact fitting of body and cap.

### 3.3. Osmotic Release Study

**Figure 7:**
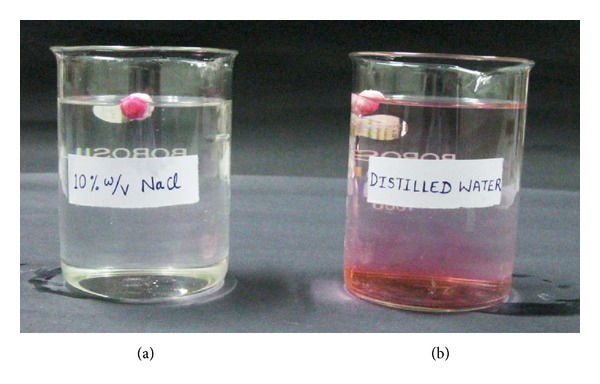
Comparative erythrosine dye release behavior from the AMCs in distilled water (b) and 10% NaCl solution (a).

A stream of the dye release was observed from the capsule suspended in distilled water after a lag time of 5 minutes, suggesting *in situ* pore formation. However, no stream of dye was observed from the capsule placed in 10% *w/v* solution of sodium chloride (Figure 7). This may be attributed to the fact that the osmotic release from the system was inactivated by the higher osmotic pressure of the surrounding medium, which did not allowed the system to release the dye. By this it can be concluded that the prepared system follows the osmotic principle for releasing the encapsulated materials [13].

### 3.4. Scanning Electron Microscopy (SEM)

**Figure 8:**
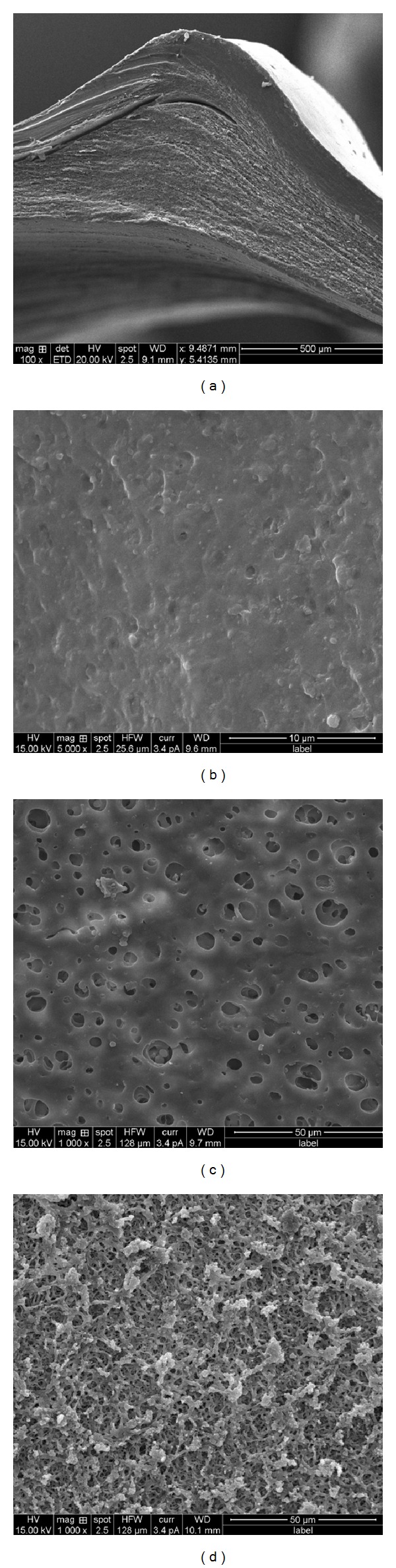
SEM images of (a) cross section, (b) surface view of CAB-12% *w/v*, PG-10% *v/v*, (c) surface view of CAB-12% *w/v*, PG-15% *v/v*, and (d) surface view of CAB-12% *w/v*, PG-20% *v/v*.

CAB-12 AMCs with varied proportions of pore forming agent, PG, were studied by SEM which revealed a distinct porous inner structure and a dense outer surface (Figure 8(a)). Increase in size and number of pores was observed in the AMCs with higher concentrations of PG which is attributed to its solubility during quenching. Thus, it can be concluded that the concentration of PG had shown positive effect on the porous nature of the AMCs (Figures 8(b), 8(c), and 8(d)).

### 3.5. Performance Evaluation of the Semiautomatic Manufacturing Process

**Figure 9:**
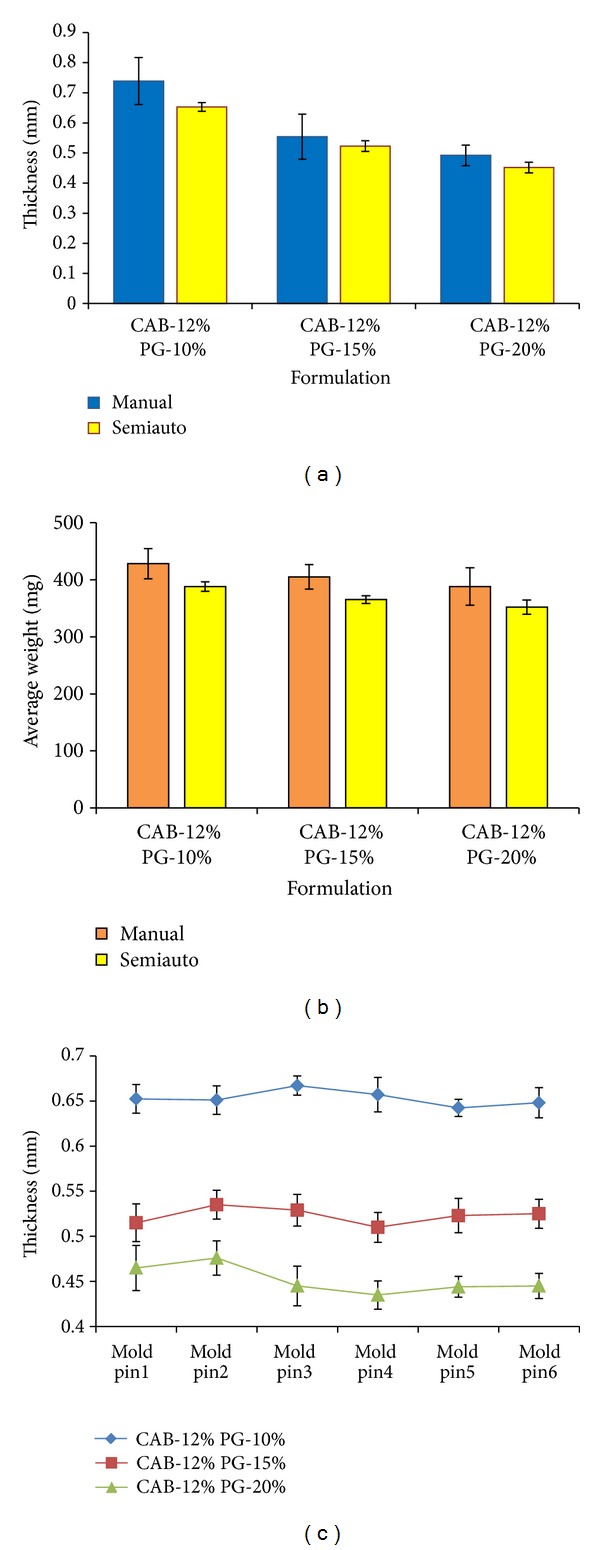
(a) Comparison of thickness, (b) weight variation between manual and semiautomatic process (*n* = 3) and (c) Variation in the thickness between individual mold pins (*n* = 3).

The efficiency of the fabricated equipment was analyzed by comparative evaluation of physical parameters in manual and semiautomatic process. CAB-12 formulation at varied levels of plasticizer (PG) was selected for the validation process. The physical parameters like thickness, weight variation, and reproducibility and variations between individual mold pins were studied. This analysis deduced the fact of slight reduction in thickness and average weight values (Figures 9(a) and 9(b)) of the AMCs prepared by semiautomatic process to manual procedure, but significant reduction in the deviation was also observed, which revealed the fact of efficiency and reproducibility of the fabricated equipment. It was also observed that there was no significant variability between the thickness difference between individual mold pins (*P* < 0.05) (Figure 9(c)). From the data it can be concluded that fabricated equipment would be a better alternative to the manual manufacturing process of the AMCs.

### 3.6. Preparation and Characterization of the Plain and Asymmetric Membranes

#### 3.6.1. FTIR Analysis

**Figure 10:**
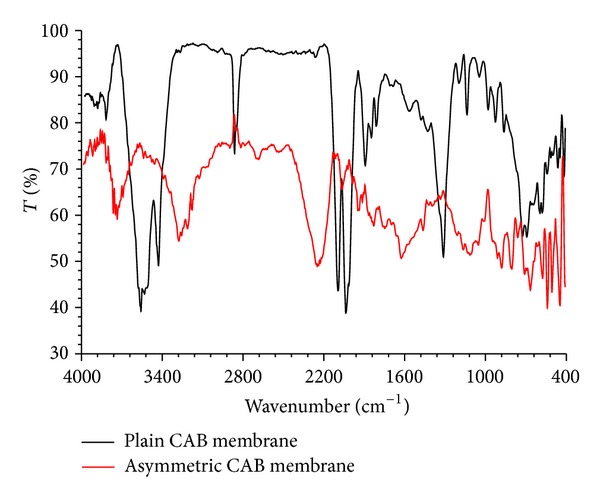
FTIR spectra of plain and asymmetric membranes.

FTIR spectra of plain and asymmetric membrane films were recorded to investigate the molecular changes due to phase inversion in CAB membranes. The spectrum obtained for plain CAB film shows broadband at 3357 cm^−1^ due to the stretching of –OH group and sharp peaks at 1623 cm^−1^ and 1093 cm^−1^ ascertained to be stretching frequency of –C=O and –C–O–C groups of CAB. However, the asymmetric membrane showed interesting molecular changes due to its significant shift in stretching frequencies. The broad band of plain CAB film at 3357 cm^−1^ was shifted to 2948 cm^−1^ and –C=O stretching peak at 1623 cm^−1^ was shifted to 1696 cm^−1^ and 1745 cm^−1^ respectively. The sharp peak of 1093 cm^−1^ observed in plain membrane was disappeared in the asymmetric membrane (Figure 10). These shifts in the stretching frequencies of asymmetric membranes confirm the fact of CAB-CAB intramolecular hydrogen bonding during phase inversion [14, 15].

#### 3.6.2. Water Vapor Transmission Rate

**Figure 11:**
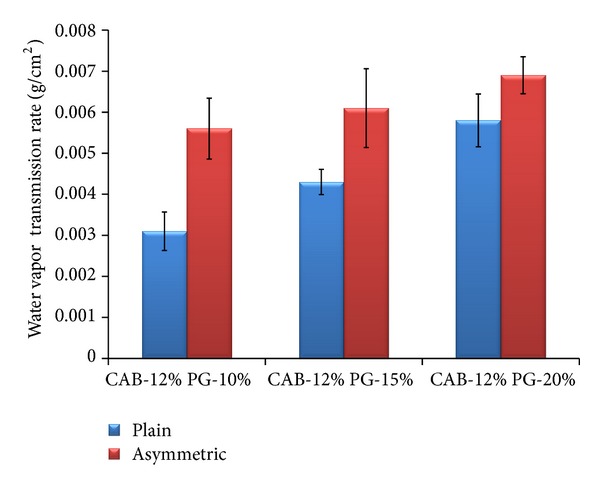
Water vapor transmission rate of plain and asymmetric membranes.

Water vapor permeability of plain and asymmetric membrane films was determined by means of water vapor transmission rate (WVTR) and the results are shown in Figure 11. The WVTR was found to be more in asymmetric membranes compared to plain membranes. The concentration of the pore forming agent had a significant positive effect on the WVTR in the asymmetric membranes. This could be due to high hydrophilic nature of PG which leads to porous nature of the asymmetric membrane [16].

#### 3.6.3. *In Vitro* Release Studies

**Figure 12:**
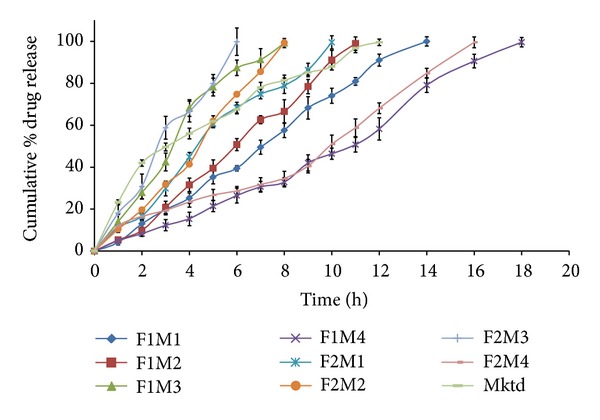
Comparative *in vitro* drug release profiles.

*In vitro* drug release studies were performed according to the factorial design batches and the results showed (Figure 12) significant difference in the release rates. The release rate of metformin hydrochloride was found to be controlled over a period of 6–18 h (Table 3). The effect of pore forming agent on the drug release was analyzed in AMCs having higher (F2M1–F2M4) and lower levels (F1M1–F1M4) of PG. The formulations with higher levels of PG showed faster drug release than those with lower levels of PG, which may be attributed to increased pore formation during the dissolution. Similarly, the total concentration of the osmogents present in the formulation had also shown cumulative effect on the drug release.

The results concluded that, when osmogent and pore former were at higher levels (F2M3), faster drug release was observed than at lower levels (F1M4). Whereas the drug release from the remaining formulations had shown the intermediate drug release patterns based on the concentrations of the osmogents and pore former.

#### 3.6.4. Kinetics of Drug Release

The release profiles of all the formulations were fitted in different models and the results showed that the best fit models for most of the formulations were the zero order and Peppas (Table 4). The formulations, F1M1, F2M3, and F2M4 were fit to zero-order kinetics and other formulations F1M2, F1M3, F1M4, F2M1, and F2M2 were found to be following Peppas model kinetics of drug release. The highest coefficient of determination *r* ^2^ ≥ 0.995 was identified for F1M1 for zero-order fit, suggesting controlled release.

#### 3.6.5. Statistical Analysis

The results of *in vitro* data were analyzed by Design Expert and it was observed that the selected independent variables (concentration of PG and amount of potassium chloride and fructose) significantly influenced the cumulative drug release from the AMCs which was evident from Table 3.

**Figure 13:**
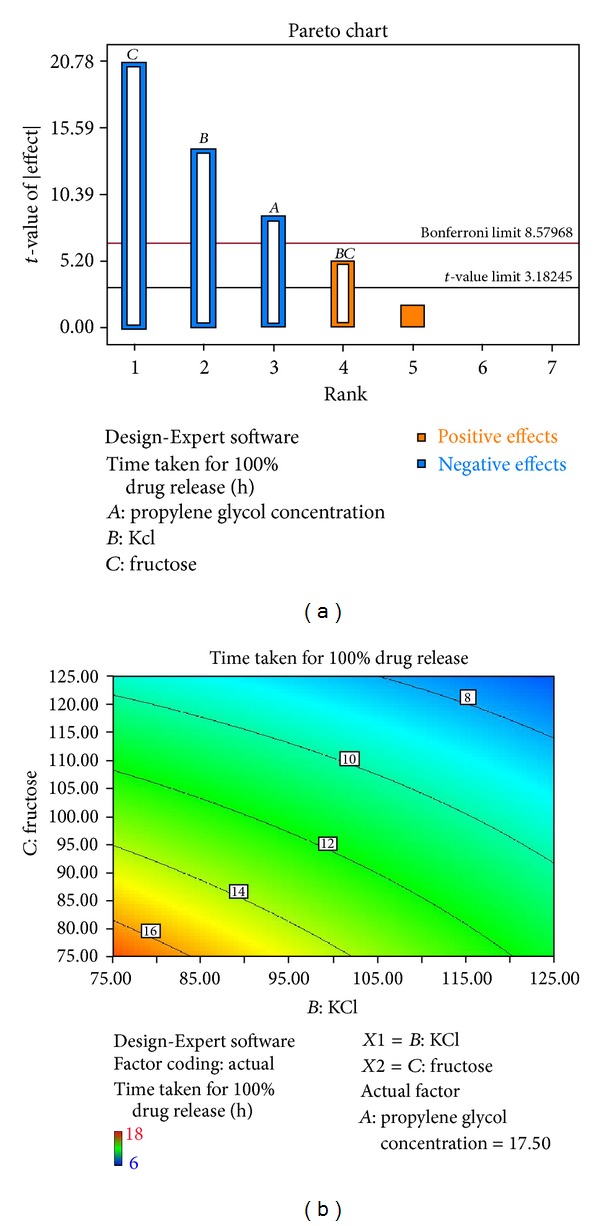
(a) Pareto chart showing the percentage contribution, (b) two factor interactions significant independent variables (*BC*).

Based on the results obtained, the response polynomial coefficients were determined in order to evaluate the response (time taken for 100% drug release, *t*_100%_). The response was studied for statistical significance by Pareto chart as shown in Figure 13(a) and the *t*-value of effect was studied by two limit lines, namely, the Bonferroni limit line (*t*-value of effect = 6.579) and *t*-value limit line (*t*-value of effect = 3.182). Coefficients with *t*-value of effect above Bonferroni line are designated as certainly significant coefficients, and coefficients with *t*-value of the effect between Bonferroni line and *t* limit line are termed as coefficients likely to be significant, while *t*-value of effect below the *t* limit line is statistically insignificant and should be removed from the analysis [17]. In the present study, the percentage contribution of independent factors (*A*, *B*, and* C*) has shown significant contribution towards the system and the combined effect of the *BC* has also shown an intermediate effect which was observed above the *t*-value limit line.

According to the percentage contribution of each variable on the response coefficients the two factor interactions of *AB* and *AC* were excluded from the analysis and the two factor interaction of *BC *was investigated (Figure 13(b)). The polynomial equation which represents simultaneous effect of any two variables on the response parameter (*t*_100%_) taking one variable at constant level was generated. Consider the following:

(2) $$\begin{array}{c} t_{100\%}\left(\text{h}\right)=11.25-1.25A-2B-3C+0.75BC. \end{array}$$

Following conclusions can be drawn from the data of rank order contribution, contour plots, and response surface graphs.

Typically, in the polynomial equation, a positive sign represents a synergistic effect, while a negative sign indicates an antagonistic effect on the system.

**Figure 14:**
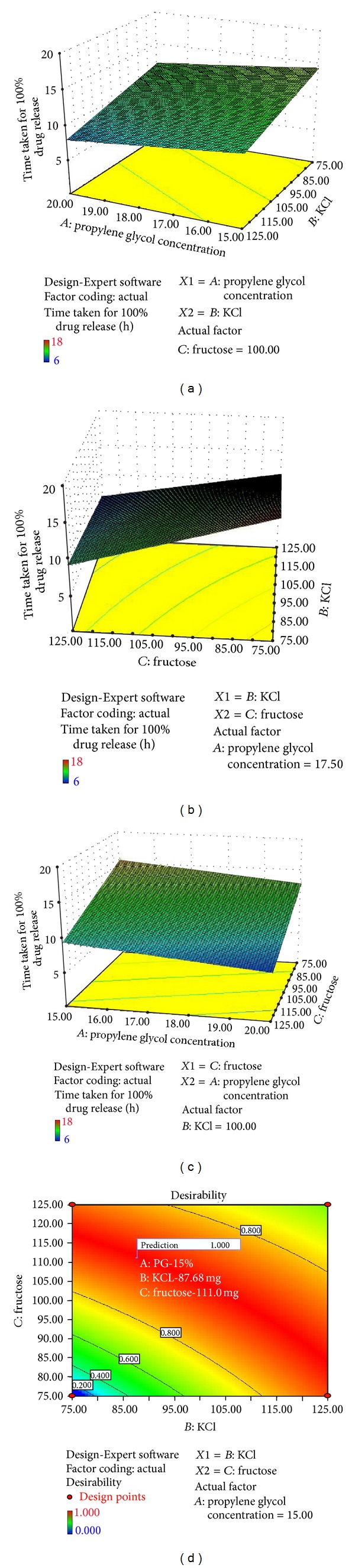
Response surface plots showing the effects of independent variables (a) *AB*, (b) *BC*, (c) *AC* and (d) contour plot showing the predicted response of the selected optimized formulation.

(1) The concentrations of the potassium chloride (*B*) and fructose (*C*) were found to be the major factors which had a direct effect on the response (*t*_100%_). The fact that osmotic pressure created inside the AMCs directly dependent on the concentration of the osmogents and combined effect of these two variables attributed their optimization on the intended response factor (*t*_100%_).

(2) The concentration of the PG (*A*) was found to be the third major contributory factor which has direct effect on response. The fact that porosity of the AMCs directly dependent on the concentration of the PG in which higher porosity leads to the faster drug release with a lower contribution of osmosis and higher contribution of diffusion. Thus, the lower concentration of the PG was recommended to get a controlled release with osmosis as a major mechanism of drug release.

(3) From the response surface graphs it was observed that increase in the concentrations of PG and potassium chloride had a negative impact on the response (*t*_100%_) (Figure 14(a)) and a medium level of interaction was observed between the factors *B* and *C* (potassium chloride and fructose) in which drug release was found to be controlled at lower levels of these two variables. The increase in the concentration of potassium chloride at a constant level of fructose had shown a minimal influence on the time taken for drug release (Figure 14(b)).

(4) From Figure 14(c), the major contribution of the fructose on the drug release was observed at higher concentrations of PG, which leads to the faster drug release. A linear effect of these two variables was observed on the response without any significant interaction.

### 3.7. Selection and Validation of the Optimized Formulation (OPT)

By fixing the response factor (*t*_100%_) as 12 h, the optimized formulation was selected among the generated solutions, of minimum PG concentration (15% *v/v*) and desirability value near to 1 (Figure 14(d)). The validation of the OPT was performed by comparing the predicted and experimental response. The *in vitro* drug release studies of the OPT showed complete drug release at the end of 13 h with zero-order kinetics with *r* ^2^ and *k* values of 0.99 and 7.89 and *n* value of 0.98. From the data, it was evident that the optimization criteria matched the experimental response at 5% level of significance.

### 3.8. Effect of pH and Agitation Intensity on Drug Release

**Figure 15:**
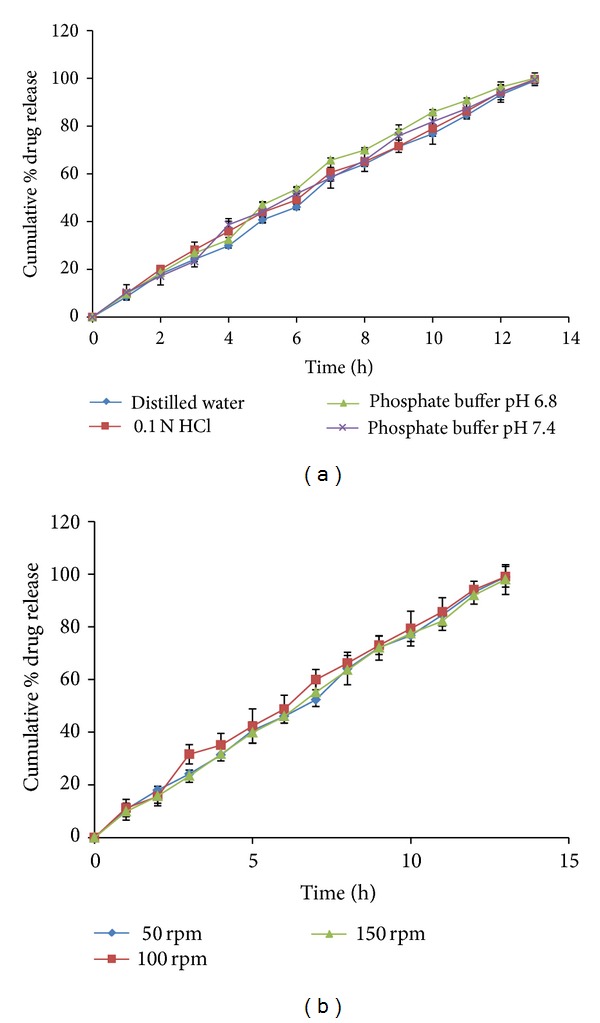
Effect of (a) pH and (b) agitation intensities on the drug release of OPT.

The release study of the OPT conducted at different pH conditions (1.2, 6.8, and 7.4) and agitation intensities (50, 100, and 150 rpm) deduced the nondependence of these parameters on drug release behavior as shown in Figures 15(a) and 15(b). These results support the fact that drug release from AMCs was probably due to the entry of the dissolution medium into the formulation which in turn was controlled by barrier layer (CAB) but not due to the pH and turbulence of the dissolution medium.

### 3.9. Effect of Osmotic Pressure

**Figure 16:**
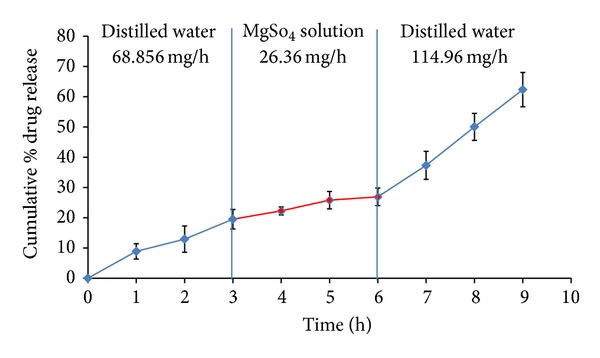
Effect of osmotic pressure on the drug release of the optimized formulation (OPT).

The release study of the OPT conducted at different osmotic environments revealed the importance of osmotic pressure on the drug release (Figure 16). Significant amount of drug release was observed at 0–3 h (68.85 mg/h) and 6–9 h (114.96 mg/h) in distilled water compared to 3–6 h (26.36 mg/h) in magnesium sulphate solution. Thus, it can be concluded that the primary mechanism of drug release from the developed system was osmotically governed.

## 4. Conclusion

A semiautomatic manufacturing process was successfully developed for the preparation of AMCs with an output of 80–100 capsules per day. The physical parameters of the capsule shells were more consistent and reproducible in semiautomatic process compared to manual process. The developed system was able to control metformin hydrochloride release for an extended period of time and the process variables were successfully optimized to control the release over a period of 13 h by osmotic mechanism. The developed system was independent of external factors like pH and agitation intensity. The process employed in the preparation was simple, makes use of limited adjuvants, and was cost effective and industrially feasible. This could be advantageous in the development of blank AMCs of consistent quality as generic osmotic delivery systems independent of drugs in relatively less time with more drug excipient combinations.

## Figures and Tables

**Table 1:** Formulation composition of AMCs of CAB.

Formulation code	Ingredients			
	CAB (% *w*/*v*)	PG (% *v*/*v*)	Ethanol (% *v*/*v*)	Acetone (% *v*/*v*)
CAB-10% PG-10%	10	10	30	70
CAB-10% PG-15%	10	15	30	70
CAB-10% PG-20%	10	20	30	70
CAB-12% PG-10%	12	10	30	70
CAB-12% PG-15%	12	15	30	70
CAB-12% PG-20%	12	20	30	70
CAB-14% PG-10%	14	10	30	70
CAB-14% PG-15%	14	15	30	70
CAB-14% PG-20%	14	20	30	70
CAB-16% PG-10%	16	10	30	70
CAB-16% PG-15%	16	15	30	70
CAB-16% PG-20%	16	20	30	70

CAB: cellulose acetate butyrate; PG: propylene glycol.

**Table 2:** Levels of independent variables taken for optimization of metformin hydrochloride formulations.

Independent variables	Levels used	
	Low	High
*A*—propylene glycol (plasticizer) (% *v*/*v*)	15	20
*B*—potassium chloride (osmogent) (mg)	75	125
*C*—fructose (osmogent) (mg)	75	125
Metformin.HCl (mg)	500	
Purified talc (mg)	3	
Magnesium stearate (mg)	2	

**Table 3:** Experimental design summary of the metformin hydrochloride formulations.

S. No	Formulation code	Independent variables			Dependent variable
		A Conc. of PG (% *v*/*v*)	B Conc. of KCl (mg)	C Conc. of Fructose (mg)	Time taken for 100% drug release (t_100%_)
1	F1M1	−1	+1	−1	8
2	F1M2	−1	−1	+1	16
3	F1M3	−1	+1	+1	8
4	F1M4	−1	−1	−1	10
5	F2M1	+1	+1	−1	11
6	F2M2	+1	−1	+1	18
7	F2M3	+1	+1	+1	6
8	F2M4	+1	−1	−1	13

(Actual values: *A*, +1 = 20% *v*/*v*, −1 = 15% *v*/*v*; *B*, +1 = 125 mg, −1 = 75 mg; *C*, +1 = 125 mg, −1 = 75 mg).

**Table 4:** Comparative drug release kinetics for the design formulations.

Form. code	Kinetic Models										Korsmeyer peppas parameter n	Best fit model
	Zero-order plot		First-order plot		Higuchi plot		Korsmeyer-peppas		Hixson-crowell			
	*r* ^2^	k	r^2^	k	r^2^	k	r^2^	k	r^2^	k		
F1M1	**0.995**	**7.414**	0.899	−0.145	0.900	20.784	0.994	4.365	0.949	−0.037	1.252	Zero-order
F1M2	0.992	8.786	0.775	−0.243	0.892	23.547	**0.995**	**4.579**	0.903	−0.051	1.315	Peppas
F1M3	0.973	13.856	0.956	−0.363	0.948	32.762	**0.987**	**14.543**	0.987	−0.082	0.988	Peppas
F1M4	0.993	4.589	0.967	−0.061	0.896	12.860	**0.996**	**3.909**	0.978	−0.018	1.062	Peppas
F2M1	0.988	10.341	0.896	−0.247	0.938	27.000	**0.988**	**10.069**	0.967	−0.057	1.021	Peppas
F2M2	0.993	11.054	0.840	−0.234	0.912	25.665	**0.997**	**9.740**	0.919	−0.057	1.066	Peppas
F2M3	**0.992**	**16.749**	0.856	−0.445	0.945	34.387	0.991	17.602	0.945	−0.097	0.970	Zero-order
F2M4	**0.971**	**5.188**	0.931	−0.073	0.910	14.752	0.963	9.775	0.950	−0.021	0.681	Zero-order
Mktd	0.8848	9.822	0.843	−0.319	**0.997**	**28.862**	0.992	25.832	0.968	−0.062	0.556	Matrix
